# The Story of the Insane from Year to Year

**Published:** 1900-10-20

**Authors:** 


### THE STORY OF THE INSANE FROM YEAR TO YEAR.

(Twelfth Series.)

### THE LINCOLNSHIRE COUNTY ASYLUM AT BRACEBRIDGE.

In this asylum the number of patients on January 1st, 1898, was 710, and on the last day of the year it had risen to 724. There were 174 admissions, 27 re-admissions, 79 recoveries, and 80 deaths. The recoveries were 39·9 per cent. of the admissions, and the deaths were 11·3 per cent. on the average number resident. Both of these percentages are above the county asylum average. Of the year's deaths, 30 were caused by cerebral and spinal diseases, 14 by consumption, 7 by pneumonia, 1 by bronchitis, and 3 by enteritis. Turning to table 10, we find that moral causes account for the insanity in 28 of the admissions, intemperance in drink in 26, hereditary influence in 54, and 22 were congenital. Among the admissions for 1898 were 6 criminal patients sent from the prison in Lincoln; and again Dr. Torney points out "the evil effect these criminal patients have upon the other inmates, and it is within my knowledge from information received from a criminal patient who was discharged recovered only a few days ago that another criminal, at present an inmate, had endeavoured to persuade this man to organise a conspiracy to attack the attendants in his ward, and also the medical superintendent." Parallel cases are known to almost every asylum medical officer, and various attempts have been made to change the system, but without success. The last great Lunacy Act contained much nonsensical matter which ought never to have seen the light, and whose place might well have been taken by a clause or two relating to these criminal lunatics, and another fixing pensions to asylum officials. The Lincolnshire asylum at present contains 52 patients more than it can properly accommodate, and Dr. Torney points out "the urgent necessity of taking a large and liberal-minded view of the present situation." The asylum would certainly bear extension up to 900 beds, and it is to be hoped that the report for 1899 will show that steps have been taken to carry these additions out. Procrastination can only mean waste of public money, because every patient boarded out in some other asylum (even if room can be obtained) will cost 5s. a week more than at Bracebridge; so that if 100 patients were disposed of under contract, it would be equal to a loss to the county of £1,300 a year. The committee "are pleased to be able to report that the medical superintendent and his assistants have attended to their duties with entire satisfaction." The weekly rate is a little under 9s. per head.

